# Building behaviors, one layer at a time

**DOI:** 10.7554/eLife.46375

**Published:** 2019-04-04

**Authors:** Claire Wyart, Vatsala Thirumalai

**Affiliations:** 1 Institut du Cerveau et de la Moelle EpinièreSorbonne UniversitéParisFrance; 2 National Centre for Biological SciencesTata Institute of Fundamental ResearchBangaloreIndia

**Keywords:** hindbrain, V2a interneurons, behavioral modules, locomotion, development, spinal cord, Zebrafish

## Abstract

New interneurons are added in the hindbrain to support more complex movements as young zebrafish get older.

**Related research article** Pujala A, Koyama M. 2019. Chronology-based architecture of descending circuits that underlie the development of locomotor repertoire after birth. *eLife* **8**:e42135. doi: 10.7554/eLife.42135

Most animals are born with an immature nervous system and, at first, they are only equipped with the rudimentary reflexes required to ingest food or avoid predators. As the nervous system matures, the complex movements needed for hunting or mating get added to the repertoire of the animals. However, it is not clear how these new motor skills are gradually acquired while the nervous system is still under construction.

This question remains challenging because it is difficult to estimate when the neurons that underpin specific behaviors are born. Now, in eLife, Avinash Pujala and Minoru Koyama of the Janelia Research Campus report that, in the hindbrain of zebrafish larvae, the birthdate of neurons determines in which type of movements these cells participate (Pujala and Koyama, 2019).

Zebrafish larvae are widely used as model organisms to study how the nervous system develops. This is because these fish are transparent, they grow outside of the mother’s body, and a wide range of genetic techniques is available to study them. Two-day-old zebrafish larvae are mostly unable to move: however, they show strong and fast escape responses when exposed to acoustic or mechanical stimuli, and they produce slow but powerful struggling movements to free themselves if they are restrained. At five days, these behaviors persist but the larvae also start to produce comparatively weaker and slower tail bends that allow them to search their environment for food.

To explore how the nervous system develops in zebrafish, Pujala and Koyama focused on V2a interneurons, a group of excitatory neurons essential for locomotion. A subset of V2a neurons is present in the hindbrain and connects to neurons in the spinal cord (Kimura et al., 2013; Kinkhabwala et al., 2011). The Janelia team then combined three methods: age-dependent photoconversion of fluorescent proteins to time the birth of the hindbrain V2a interneurons; paired-electrophysiological recording to map their connectivity; and population recording to monitor their activity during movement. These experiments showed that V2a interneurons developed in the hindbrain to implement the new, refined movements. Meanwhile, early-born neurons continued to underpin the old and crude reflexes of escapes and struggles (Figure 1). Further experiments revealed that the birth order of the cells determined their biophysical properties and how they connected to spinal neurons, thus allowing V2a interneurons born at different times to support distinct behaviors.

**Figure 1.**
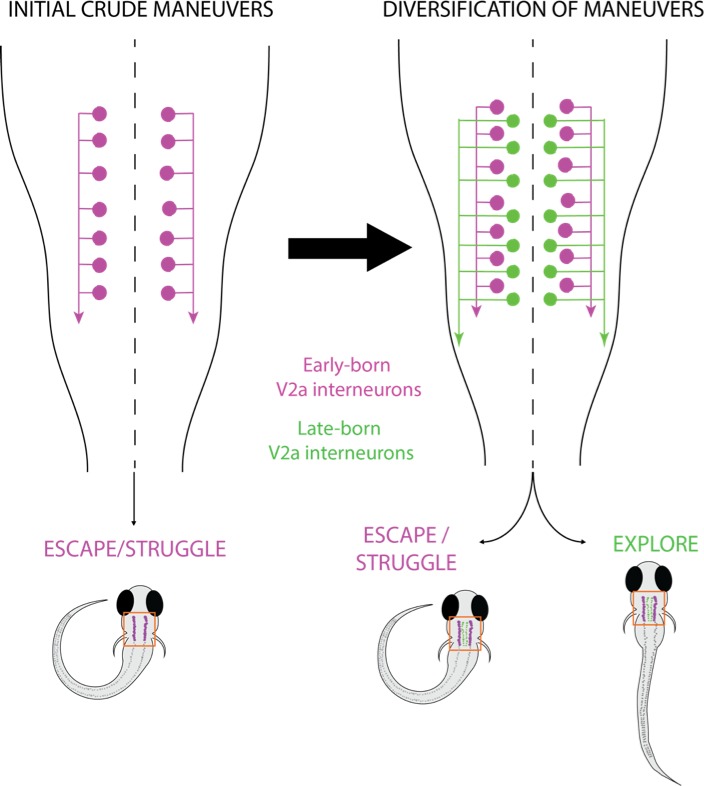
Parallel layers of interneurons in zebrafish larvae. In two-day-old zebrafish larvae (left), early-born V2a interneurons (purple) in the hindbrain are recruited to circuits to produce rudimentary movements such as escape and struggle. In five-day-old larvae (right), a parallel layer of late-born V2a interneurons (green) is added to the existing circuits to support a range of complex movements, such as spontaneous slow swimming to search for food.

These experiments build on previous studies which demonstrated that, in zebrafish, the birthdate of premotor interneurons in the spinal cord determines which locomotion pattern these cells control (McLean et al., 2008; McLean et al., 2007). The study by Pujala and Koyama also extends to the hindbrain the concept of behavioral ‘modules’. These units rely on subcircuits of neurons that are recruited based on the speed and vigor required by movements (Ampatzis et al., 2014; Song et al., 2018).

It was already known that new motor behaviors can emerge by reconfiguring existing circuits through changes in connectivity or the addition of neuromodulatory projections (Marder and Rehm, 2005; Marin-Burgin et al., 2008). For instance, neuromodulators such as dopamine can act on spinal or supraspinal targets, making motor patterns mature during development (Lambert et al., 2012; Thirumalai and Cline, 2008). What the study by Pujala and Koyama demonstrates is that new circuits can form alongside existing components, which helps to expand the repertoire of movements.

Why would organisms benefit from such a layered organization? First, gradually adding new circuits might be an efficient way to create modules that generate movements of varying speed and vigor; it might also help gate transitions between such modules. Different circuits may then be recruited to generate appropriate motor patterns in response to immediate needs.

Second, acquiring these parallel circuits one after the other helps to preserve the ‘old’ modules required for survival, even as many new neurons and connections appear in the central nervous system. Finally, this parallel organization allows new circuits and behaviors to be added in an open-ended manner, without imposing constraints based on existing circuits.

Many mechanisms, including the addition of new neurons, preside over the acquisition of brain functions such as language and memory. In the long run, the work by Pujala and Koyama provides a framework in which to investigate these processes.

## References

[bib1] Ampatzis K, Song J, Ausborn J, El Manira A (2014). Separate microcircuit modules of distinct V2a interneurons and motoneurons control the speed of locomotion. Neuron.

[bib2] Kimura Y, Satou C, Fujioka S, Shoji W, Umeda K, Ishizuka T, Yawo H, Higashijima S (2013). Hindbrain V2a neurons in the excitation of spinal locomotor circuits during zebrafish swimming. Current Biology.

[bib3] Kinkhabwala A, Riley M, Koyama M, Monen J, Satou C, Kimura Y, Higashijima S, Fetcho J (2011). A structural and functional ground plan for neurons in the hindbrain of zebrafish. PNAS.

[bib4] Lambert AM, Bonkowsky JL, Masino MA (2012). The conserved dopaminergic diencephalospinal tract mediates vertebrate locomotor development in zebrafish larvae. Journal of Neuroscience.

[bib5] Marder E, Rehm KJ (2005). Development of central pattern generating circuits. Current Opinion in Neurobiology.

[bib6] Marin-Burgin A, Kristan WB, French KA (2008). From synapses to behavior: development of a sensory-motor circuit in the leech. Developmental Neurobiology.

[bib7] McLean DL, Fan J, Higashijima S, Hale ME, Fetcho JR (2007). A topographic map of recruitment in spinal cord. Nature.

[bib8] McLean DL, Masino MA, Koh IY, Lindquist WB, Fetcho JR (2008). Continuous shifts in the active set of spinal interneurons during changes in locomotor speed. Nature Neuroscience.

[bib9] Pujala A, Koyama M (2019). Chronology-based architecture of descending circuits that underlie the development of locomotor repertoire after birth. eLife.

[bib10] Song J, Dahlberg E, El Manira A (2018). V2a interneuron diversity tailors spinal circuit organization to control the vigor of locomotor movements. Nature Communications.

[bib11] Thirumalai V, Cline HT (2008). Endogenous dopamine suppresses initiation of swimming in prefeeding zebrafish larvae. Journal of Neurophysiology.

